# 3D Reconstruction and Restoration Monitoring of Sculptural Artworks by a Multi-Sensor Framework

**DOI:** 10.3390/s121216785

**Published:** 2012-12-06

**Authors:** Sandro Barone, Alessandro Paoli, Armando Viviano Razionale

**Affiliations:** Department of Mechanical, Nuclear and Production Engineering, University of Pisa, Largo Lucio Lazzarino, n.1, 56126 Pisa, Italy; E-Mails: s.barone@ing.unipi.it (S.B.); a.razionale@ing.unipi.it (A.V.R.)

**Keywords:** 3D imaging sensors, stereo-photogrammetry, structured light scanning, cultural heritage, 3D reference information system

## Abstract

Nowadays, optical sensors are used to digitize sculptural artworks by exploiting various contactless technologies. Cultural Heritage applications may concern 3D reconstructions of sculptural shapes distinguished by small details distributed over large surfaces. These applications require robust multi-view procedures based on aligning several high resolution 3D measurements. In this paper, the integration of a 3D structured light scanner and a stereo photogrammetric sensor is proposed with the aim of reliably reconstructing large free form artworks. The structured light scanner provides high resolution range maps captured from different views. The stereo photogrammetric sensor measures the spatial location of each view by tracking a marker frame integral to the optical scanner. This procedure allows the computation of the rotation-translation matrix to transpose the range maps from local view coordinate systems to a unique global reference system defined by the stereo photogrammetric sensor. The artwork reconstructions can be further augmented by referring metadata related to restoration processes. In this paper, a methodology has been developed to map metadata to 3D models by capturing spatial references using a passive stereo-photogrammetric sensor. The multi-sensor framework has been experienced through the 3D reconstruction of a Statue of Hope located at the English Cemetery in Florence. This sculptural artwork has been a severe test due to the non-cooperative environment and the complex shape features distributed over a large surface.

## 1. Introduction

Technological developments in 3D imaging have recently allowed innovative applications for conservation, reproduction, study and fruition of sculptural, architectural and archaeological artworks [1–3]. Cultural Heritages may greatly benefit from 3D digital reconstructions, which can be used to monitor both degradation phenomena and restoration activities, or to archive multimedia representations to be used for virtual reality systems [4]. The three-dimensional digitalization of an artwork typically requires the reconstruction of complex shapes with small details over large dimensions. However, great sizes and small geometrical details are usually conflicting attributes within a shape digitalization process.

Optical techniques allow the reconstruction of artwork shapes by aligning high resolution range maps captured from different views into a common reference system [5–8]. The crucial problem in combining multiple views is the computation of the best transformation parameters (translation and rotation), which relate range maps to a common reference system [9].

The integration between 3D optical sensors and mechanical devices (*i.e.*, turntables, robotic arms, coordinate measurement machines) could be a valid solution to the automatic alignment of large data sets [10,11]. However, the mechanical devices typically lack in flexibility due to restrictions in size and complexity of the measureable shapes. Moreover, these systems are hardly portable for *in situ* measurement activities.

Alternatively, the multi-view alignment can be automatically performed by measuring the 3D coordinates of physical references, which can be either distributed over the scene or rigidly connected to the optical sensor. The distribution of fixed targets on the background generally requires a preliminary planning of the reconstruction strategy. This approach could limit the flexibility of the measurement process, since the working environment is not always suitable for a proper distribution of physical references [12,13]. The reference-based approach could be efficiently improved by physically connecting the references to the range map sensor in order to progressively track its spatial placements by an independent vision system [14]. Although some industrial proposals [15,16] have provided alignment procedures based on combining two imaging sensors, their extension to Cultural Heritage still requires further researches focused on *in situ* measurements of large sculptural artworks with small details.

This paper aims at introducing a multi-sensor framework dedicated to the Cultural Heritage field. The methodology is based on integrating two portable optical sensors: a structured light scanner and a passive stereo-photogrammetric device. In particular, high resolution range maps are acquired by a scanner based on structured lighting and encoded pattern analysis. The complete reconstruction of an artwork shape is obtained through a multi-view process consisting in capturing 3D range maps from different views and transposing them into a global coordinate system. In particular, a stereo-photogrammetric sensor automatically tracks a frame of infrared (IR) active spherical markers, which are rigidly connected to the scanner. The 3D marker coordinates are extracted by a triangulation procedure given the conjugated 2D marker stereo pairs detected on the basis of automatic intensity-based analyses. The optical tracking of the marker frame allows the range maps to be referred to the global coordinate system defined by stereo-photogrammetric sensor.

The use of an active tracking system provides a more robust methodology with comparison to the passive approach adopted in [14]. Active markers can be synchronized with the tracking system cameras yielding their prompt and unambiguous identification within the marker frame. Moreover, the use of synchronized markers allows a straightforward detection of spherical features regardless of the environmental lighting conditions.

In this paper, the 3D reconstruction methodology has been further augmented by developing a tool to monitor interventions performed on the artwork and to document the restoration activities directly on the virtual model. In particular, a **R**eference **I**nformation **S**ystem (RIS) has been developed to refer metadata regarding restoration processes, to 3D artwork representations, within a “*multi-temporal*” registry. The methodology is based on using a passive stereo-photogrammetric sensor, such as the system used to track the optical scanner, to capture spatial references, which are used to relate the points/areas of interest to the 3D model.

The multi-sensor framework effectively supports the creation of high-end Cultural Heritage models by combining automatic reconstructions of complex shapes with multiple data stored during historico-artistic and scientific activities. Moreover, the methodology allows the reduction of the long time usually needed for capturing artwork shapes by introducing automatic alignments with minimal operator’s control. The framework includes portable and configurable sensors, which allow on-site 3D digitalization, even in complex logistics.

The proposed solution has been tested by reconstructing a Statue of Hope located at the English Cemetery in Florence (Italy). This artwork has been chosen for its technical difficulties, due to the extremely complex geometrical morphology, the large dimensions and the non-cooperative working environment.

## 2. 3D Imaging Sensors

An active structured light scanner and a passive stereo photogrammetric system have been integrated in order to capture high resolution range maps and to track the measurement views within the working area, respectively. The overall measurement system has been designed aiming at configurable and cost effective layouts, which assure performances, in terms of accuracy and resolution, suitable for the Cultural Heritage applications. In particular, different measuring volumes are obtained by setting the optical parameters of the sensors through a proper selection of the lenses and the mutual disposition of the imaging devices.

### 2.1. Structured Light Scanner

**Figure 1.**
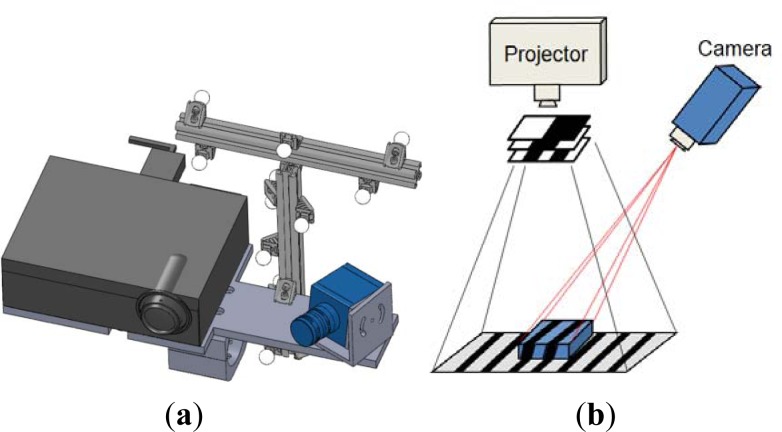
(**a**) Scheme of the 3D optical scanner. (**b**) Fringe pattern projection.

The 3D optical scanner is based on a structured lighting approach, which uses binary patterns in order to capture three-dimensional range maps. The system (Figure 1(a)) is composed of a DLP projector (1,024 × 768 pixels) to generate black and white striped light patterns and a monochrome digital CCD camera (1,280 × 960 pixels). Camera and projector are used as active devices for a stereo triangulation process. A calibration procedure is adopted to calculate the intrinsic and extrinsic parameters of the optical devices, with respect to an absolute reference system [17,18]. The projector is modeled like an inverse camera, exploiting its capability to generate both coded vertical and horizontal fringes [19].

In this work, a vertical binary encoded light stripe approach is used for 3D shape recovery. In particular, a sequence of vertical light planes is projected onto the model to be reconstructed (Figure 1(b)). The planes are defined as crossing areas between black and white parallel fringes whose period is progressively halved. Each pixel in the camera images is characterized by a temporal sequence of light intensities that can be either bright or dark depending on its location in the respective plane image. A binary code (0, 1 with *n* bit) is assigned to each pixel, where *n* is the number of the projected stripe patterns, and the values 0 and 1 are associated to the intensity levels, *i.e.*, 0 = black and 1 = white. This encoding procedure provides *l = 2^n^*−*1* encoded lines. The 3-D coordinates of the observed scene point are then computed by intersecting the optical ray with the plane considering that the geometry of the hardware set-up, the camera ray direction and the plane equation of the corresponding stripe are known. The methodology provides *n_p_ = l_h_ × l_v_* encoded points where *l_h_* is the horizontal resolution of the projector and *l_v_* is the vertical resolution of the camera.

The scanner accuracy has been tested for different working areas by measuring a reference nominal shape [19]. The experimental validation has demonstrated that the accuracy is equivalent to 10^−7^ of the field of view (FOV), whereas the lateral resolution achieved has been in the order of 10^−6^ of FOV.

### 2.2. Stereo Photogrammetric System

**Figure 2.**
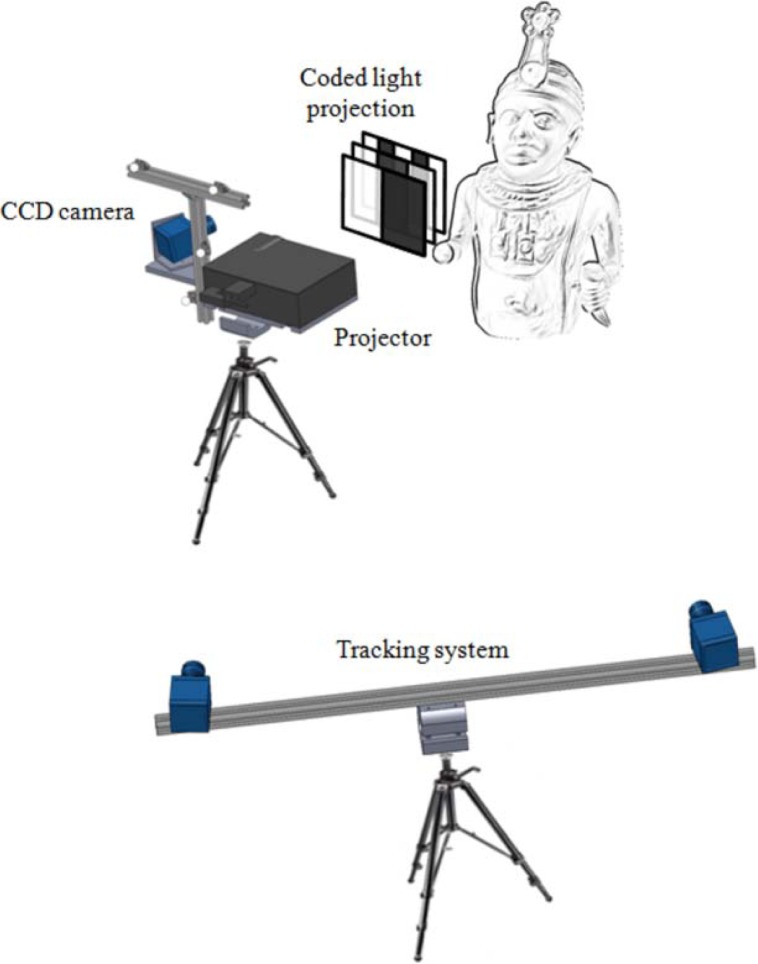
Scheme of the measurement process for a 3D artwork reconstruction.

The hardware set-up consists of a stereo rig (Figure 2) including two CMOS cameras (9 fps, resolution 2,048 × 1,536 pixels, sensibility to near infra-red light) mounted on a stand with an adjustable baseline (distance between the cameras). The CMOS cameras are equipped with 12 mm focal length lenses with infrared filters (825 nm cut-off wavelength). The optical layout can be configured in order to adapt the field of view to the measurement requirements (*i.e.*, *environmental conditions* and *working volume*). The optical configuration is calibrated by evaluating the intrinsic and extrinsic parameters of the stereo system. The optical sensor is mounted on a camera tripod, which is steady and easy to carry.

The photogrammetric sensor is used to measure the 3D coordinates of active markers, which are rigidly connected to the optical scanner (Figure 1(a)). The marker set defines a reference frame, which is characterized by the following marker parameters: spatial distribution, number, spherical size. These parameters are set to minimize occlusion problems and to optimize the scanner pose estimation. Each active marker is composed of several single LEDs (940 nm wavelength), covered by spherical diffusers which provide visibility from all viewing directions up to long distances. All the markers are synchronized with the stereo photogrammetric sensor. In particular, the markers are consecutively switched on/off, only one light at a time, thus providing an unambiguous identification within the reference frame. The use of synchronized active markers allows a robust detection of spherical features in the scene through straightforward procedures based on image processing algorithms.

## 3. Multi-View 3D Reconstruction

The complete recovery of a complex shape generally requires the acquisition of multiple range maps from different views, which have to be transformed into a global coordinate system. In this paper, the alignment process is carried out by firstly referring each range map to the reference frame of spherical markers rigidly connected to the scanner structure. Besides, the range maps are transposed to the global reference system defined by the stereo photogrammetric sensor. The methodology is composed of the following steps: (*i*) computation of 3D marker coordinates, (*ii*) calibration of the marker frame, (*iii*) pose estimation of the optical scanner.

### 3.1. Spherical Marker Identification

The first step of the marker identification process consists in detecting circular shapes from grey level images captured by the stereo-photogrammetric sensor. The synchronization between markers and cameras and the active control of the marker lighting allows each single maker to be imaged as a white circle over a dark background by simply subtracting the scene with natural illumination from the images acquired when the marker is turned on.

**Figure 3.**
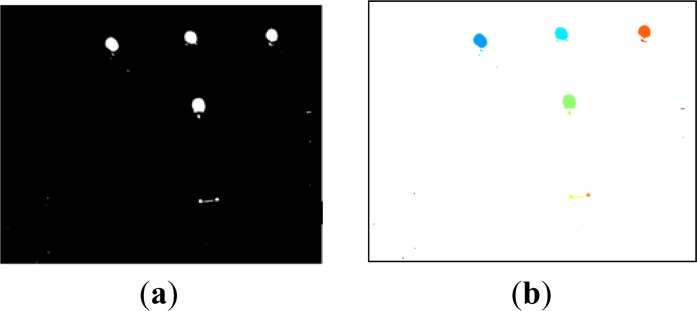
(**a**) Binarized image. (**b**) Labeled image.

The coarse circle detection is performed by binarizing the original image using a threshold value. Figure 3(a) simultaneously shows four segmented markers obtained by independently processing the respective images captured during a temporal sequence. All the connected regions resulting from the binarization process are then labeled (Figure 3(b)) and a set of two shape descriptors, area (*A_CR_*) and extent (*X_CR_*, proportion of pixels in the connected region with respect to the circumscribed rectangular box), are calculated. The shape descriptors have to simultaneously verify the following constraints:

(1) $$\{\begin{array}{c} A_{\text{min}}\leq A_{\mathrm{CR}}\leq A_{\text{max}}\\ 0.6\leq X_{\mathrm{CR}}\leq 0.8 \end{array}$$

where *A_min_* and *A_max_* are empirically defined on the basis of the image resolution, and the extent range takes into account the pixel discretization and the border effect (*X_CR_* = π/4 for a circular theoretical shape). This control is carried out in order to discard false positives due to uneven reflections occurring between the two images. Centroids of the connected regions satisfying relations (1) are then computed and refined on the grayscale difference image, over a window centered on the preliminary detected position.

The 3D marker coordinates are determined by a stereo triangulation procedure given the conjugated 2D marker pairs. The proposed methodology provides a straightforward stereo matching through the synchronization between marker lighting and camera capturing. The temporal sequence based on switching on and off the active spheres allows an unambiguous identification of different markers within the global reference frame (Figure 3(b)).

### 3.2. Marker Frame Calibration

**Figure 4.**
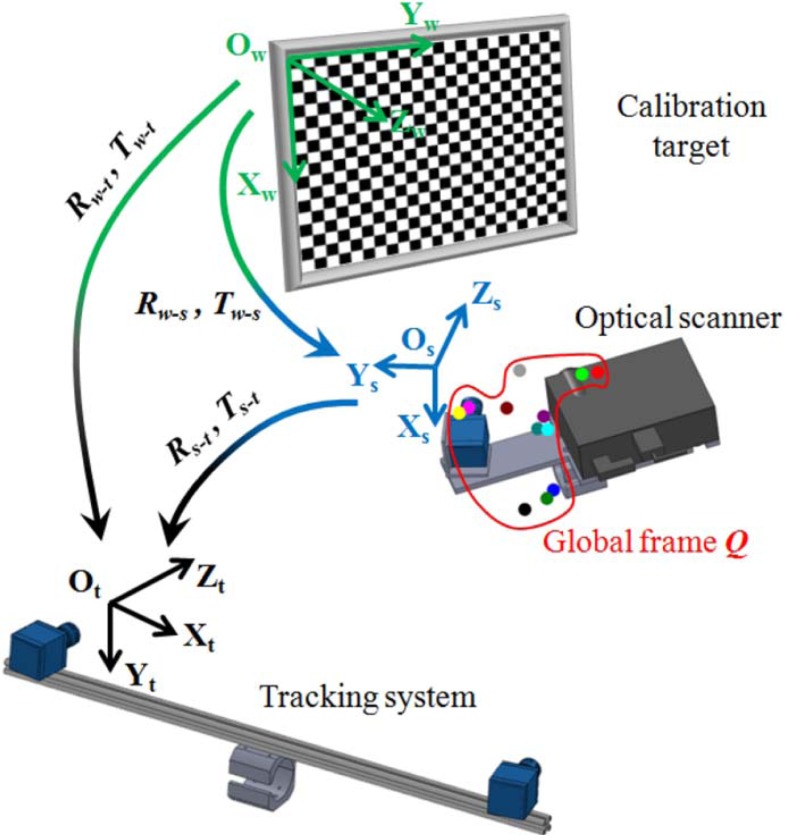
Scheme of the global markers frame calibration relating 3D markers coordinates in the optical scanner reference system. Different colors identify different spheres.

The structured light scanner provides range maps with reference to the scanner local coordinate system (O_s_, X_s_, Y_s_, Z_s_). The alignment process consists in referring each range map with respect to the coordinate system integral to the stereo photogrammetric sensor (O_t_, X_t_, Y_t_, Z_t_). This process is based on a calibration process, which consists in referring the marker frame (***Q***) composed of *M_q_* markers (with *q* ≥ 3) to the scanner system (O_s_, X_s_, Y_s_, Z_s_). The calibration process requires the use of points having known positions in a world reference system (O_w_, X_w_, Y_w_, Z_w_). In this work, a calibrated board, composed of a printed pattern onto a planar glass surface, has been used. The calibration procedure is performed by placing the board in a position where the targets are visible by both the structured light scanner and the stereo photogrammetric system (Figure 4). The 3D reconstructions of the chessboard corners are obtained both in the scanner and stereo photogrammetric systems.

The rigid body transformation (***R**_w-s_*, ***T**_w-s_*) from the world reference system, integral to the chessboard, to the scanner reference system are determined by the singular value decomposition (SVD) method applied to the respective 3D point structures. Rotation and translation parameters (***R****_w-t_*, ***T****_w-t_*) from the world reference system to the stereo photogrammetric system can be similarly obtained. The composition of the two rigid transformations determines the rotation and translation parameters (***R****_s-t_*, ***T****_s-t_*) from the scanner to the stereo photogrammetric systems. The homogeneous marker coordinates measured in the system (O_t_, X_t_, Y_t_, Z_t_) are expressed as:

(2) $$Q_{t}=({\mathrm{Qx}}_{t},{\mathrm{Qy}}_{t},{\mathrm{Qz}}_{t},1)$$

The coordinates ***Q****_t_* are transformed in the scanner reference system by applying the operators ***R****_st_* and ***T****_st_* as:

(3) $$Q_{s}=\left(\begin{array}{cc} R_{s-t}^{-1} & T_{s-t}^{-1}\\ 0 & 1 \end{array}\right)Q_{t}$$

During the calibration process, different placements of the scanner, in front of the calibration board, are used in order to handle possible occlusions and reconstruct the overall markers frame ***Q***. Different measurements of the same sphere are averaged to obtain the marker reference within the global frame. The redundant number of markers ensures that at least three spheres are always visible by the stereo photogrammetric set-up arrangement, even in cases of partial occlusions. Moreover, the substantial redundancy is employed in order to minimize the unavoidable coordinate estimation errors.

### 3.3. Pose Estimation of the Optical Scanner

**Figure 5.**
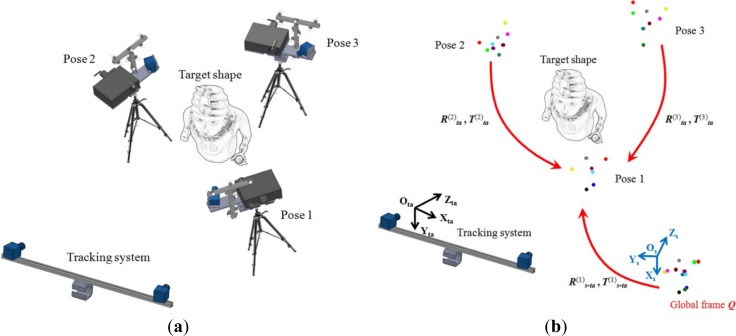
(**a**) Three different poses of the optical scanner during the measurement process. (**b**) Frame matching of common markers for the range maps alignment.

The last step of the developed tracking approach consists of estimating the rigid motions of the optical scanner in order to align the acquired range maps (Figure 5(a)). This process is carried out by identifying and matching common markers with respect to the global calibrated frame ***Q****_s_* for each range map acquisition. During the target object measurement process, each markers set ***P***, composed of *M_p_* markers (with *q* ≥ *p* ≥ 3) captured by the stereo photogrammetric sensor, is expressed in the tracking reference system (O_ta_, X_ta_, Y_ta_, Z_ta_) as:

(4) $$P_{\mathrm{ta}}^{\left(k\right)}=({\mathrm{Px}}_{\mathrm{ta}}^{\left(k\right)},{\mathrm{Py}}_{\mathrm{ta}}^{\left(k\right)},{\mathrm{Py}}_{\mathrm{ta}}^{\left(k\right)},1)$$

where *k* = 1,…,*k_i_* specifies the scanner pose. In particular, the global frame ***Q****_s_* is matched to the set ***P***^(k)^*_ta_*, captured when the scanner is placed in its first position (*k* = 1). The rigid motion (***R***^(1)^*_s-ta_*, ***T***^(1)^*_s-ta_*) between the two structures is determined by the SVD method. The homogeneous marker coordinates of the global frame ***Q****_s_* are transformed in the stereo photogrammetric system (Figure 5(b)) by applying ***R***^(1)^*_s-ta_* and ***T***^(1)^*_s-ta_*:

(5) $$Q_{\mathrm{ta}}^{\left(1\right)}=\left(\begin{array}{cc} R_{s-\mathrm{ta}}^{\left(1\right)} & T_{s-\mathrm{ta}}^{\left(1\right)}\\ 0 & 1 \end{array}\right)Q_{s}$$

For each successive scanner pose, the translation vector ***T***^(k>1)^*_ta_* and the rotation matrix ***R***^(k>1)^*_ta_*, defining the rigid motion with respect to first pose, are directly determined by applying the SVD method between common markers of the captured set ***P***^(k>1)^*_ta_* and the global marker calibrated frame ***Q***^(1)^*_ta_* expressed in the tracking reference system.

**Figure 6.**
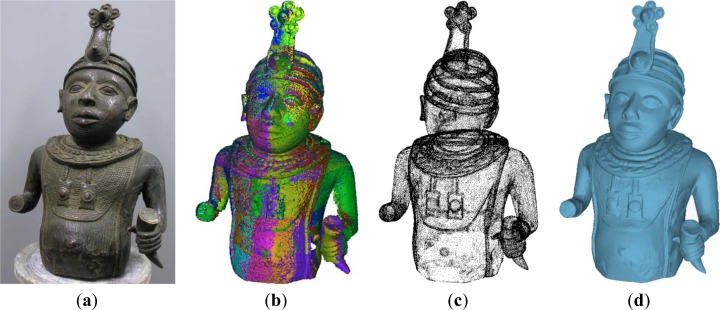
(**a**) Bronze model of an African bust. (**b**) Alignment of 48 different range maps. (**c**) Merging and sampling of the captured data. (**d**) StL final model.

**Figure 7.**
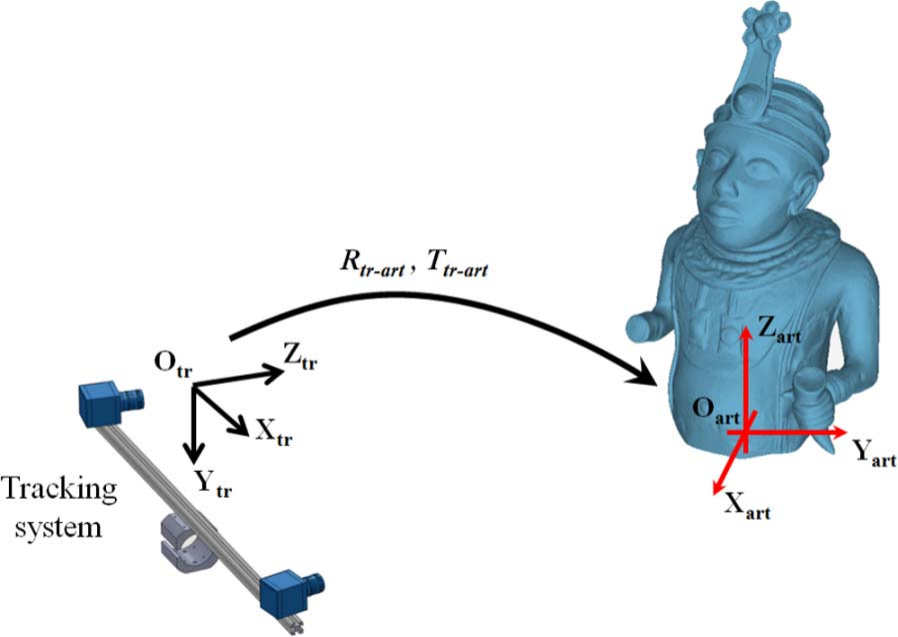
Scheme of the Reference Information System. The rigid motion (***R****_tr-art_*, ***T****_tr-art_*) is determined through a calibration process.

As example, Figure 6(a) shows a bronze model representing an African bust of unknown origin. The model has been reconstructed by coarsely aligning 48 different range maps captured through the proposed tracking methodology and refining the alignment by an Iterative Closest Point (ICP) algorithm [20] to minimize mismatches between overlapping surfaces (Figure 6(b)). The overall sample points (∼8 million) have been reduced and post processed by proper algorithms of smoothing and filtering (Figure 7(c)). The final point cloud is then triangulated to obtain the mesh representation as shown in Figure 7(d).

## 4. Reference Information System

In this paper, a methodology has been developed to refer alphanumerical information regarding degradation monitoring and restoration processes to the 3D artwork model. In particular, metadata are spatially referred to artwork 3D models by adopting an independent stereo-vision sensor. In this work, the experimental activities have been carried out by using the same stereo photogrammetric system developed to track the optical scanner. The methodology consists in:

- 3D spatial referring the stereo vision system with respect to the artwork model through a calibration process;
- selecting and detecting points/areas of interest;
- matching metadata to points/areas of interest.

The calibration procedure is carried out in order to determine the rigid motion (***R****_tr-art_*, ***T****_tr-art_*), which refers the coordinate system (O_tr_, X_tr_, Y_tr_, Z_tr_) of the independent stereo sensor with respect to a coordinate system (O_art_, X_art_, Y_art_, Z_art_) integral to the artwork virtual model (Figure 7).

The rigid motion parameters (***R****_tr-art_*, ***T****_tr-art_*) allow the 3D model to be augmented through the direct association of metadata, whose spatial address is defined by points and/or lines captured by the independent stereo sensor on the physical model. In particular, the stereo set-up is properly configured to the target information and placed in front of the working area without any spatial constraints or prior knowledge about the relative orientation with respect to the artwork.

**Figure 8.**
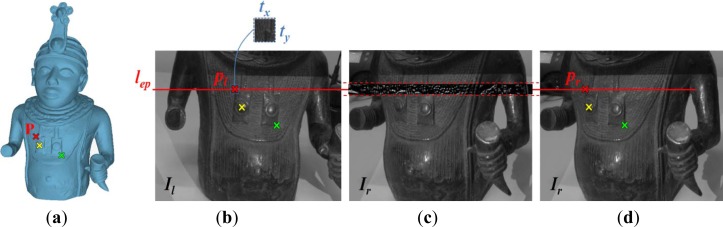
(**a**) Reference points manually selected from the 3D artwork mesh model. (**b**) Selection of 2D reference points on the left image. (**c**) Right image with superimposed the band including the cross correlation intensity values. (**d**) The conjugate points obtained by the automatic stereo matching.

The calibration process relies on the use of some reference points (at least three references), which must be easily recognizable both in the images acquired by the stereo sensor (left and right image) and on the 3D artwork model. The reference points are manually selected on the 3D model (Figure 8(a)) and on one of the stereo image (later on, the left image is used). For each reference point, the stereo matching is then automatically carried out by exploiting the epipolar constraint and the normalized cross correlation [21] between the stereo images. Operatively, stereo images are rectified by using the optical calibration parameters [22]. Rectification consists in transforming each image plane such that pairs of conjugate epipolar lines become parallel to the image axes, usually the horizontal one. In the rectified configuration, conjugate points have therefore the same vertical coordinate, within a certain tolerance. Once selected a reference point *p_l_* = (*u_l_*,*v_l_*) on the rectified left image *I_l_* (Figure 8(b)), the detection of its conjugate point *p_r_* = (*u_r_*,*v_r_*) on the rectified right image *I_r_* (Figure 8(d)) is efficiently reduced to the determination of the position of the pattern template *t* (size *t_x_* × *t_y_*) represented by a window centered in *p_l_*, within a band of *I_r_* symmetric with respect to the epipolar line *l_ep_*. Let *f*(*x*,*y*) denote the intensity value of this narrow rectangular band with size *m_x_* × 2*t_y_* (Figure 8(c)). The position of the template *t* in the image *f* can be detected by evaluating the normalized cross correlation value *γ* at each point *p* = (*u*,*v*) for the analysis image *f* and the template window *t* which is shifted by *u* steps in the *x* direction and by *v* steps in the *y* direction:

(6) $$\gamma =\frac{\sum_{x,y}\left(f(x,y)-\overset{\_}{\underset{u,v}{f}}\right)\left(t(x-u,y-v)-\bar{t}\right)}{\sqrt{\sum_{x,y}{\left(f(x,y)-\overset{\_}{\underset{u,v}{f}}\right)}^{2}\sum_{x,y}{\left(t(x-u,y-v)-\bar{t}\right)}^{2}}}$$

where:

(7) $$\overset{\_}{\underset{u,v}{f}}=\frac{\sum_{x=u}^{u+t_{x}-1}\sum_{y=v}^{v+t_{y}-1}f(x,y)}{t_{x}t_{y}}$$

denotes the mean value of *f*(*x*,*y*) within the area under the template and *t̄* is the mean of the template. The conjugate point *p_r_* is finally given by the maximum value *γ_max_* of *γ*(*u*,*v*) (Figure 8(d)). In this work, a *t_x_* = *t_y_* = 11 pixels template window has been used. Three-dimensional coordinates of the correspondent pairs of reference points have been evaluated by means of a triangulation process. The rigid motion ***R****_tr-art_*, ***T****_tr-art_* is finally determined by the SVD method applied to the respective 3D point structures.

Once the relative pose between the independent stereo system and the physical artwork model has been determined, the spatial references of the metadata are acquired by one or more stereo image pairs. Geometrical indicators (such as points, lines, and areas) can be defined by laser pointers, physical targets or shape features.

**Figure 9.**
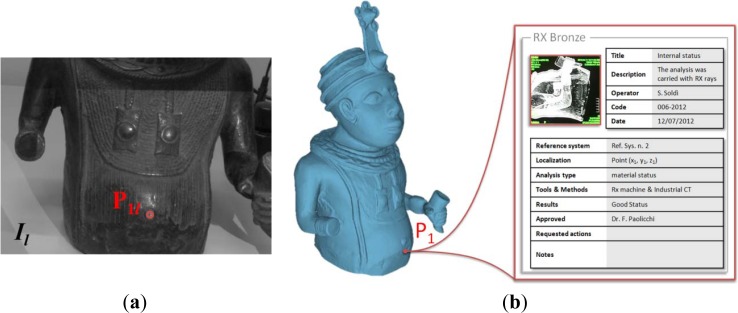
(**a**) 2D selection of a reference point on the left stereo image. (**b**) Radiographic analyses associated to the virtual model by using the correspondent 3D point.

In Figure 9(a), a reference point corresponding to a shape feature of the bronze bust is manually selected on an image of the stereo system. The corresponding 3D coordinates are transformed into the artwork reference system through the rigid motion ***R****_tr-art_*, ***T****_tr-art_*. The 3D point is orthogonally projected onto the artwork mesh model. The point projection takes into account possible inaccuracies occurring during the calibration processes. Metadata relative to restoration activities and/or status analyses of the artwork can be stored in an open “*.xml*” file, easily accessible by any information system, and directly associated to the corresponding reference onto the 3D virtual model. As example, Figure 9(b) shows the data relative to a radiographic analysis which has been spatially stored and addressed to the corresponding region of the bronze virtual model.

## 5. Three-Dimensional Reconstruction of the Statue of Hope (Florence, Italy)

**Figure 10.**
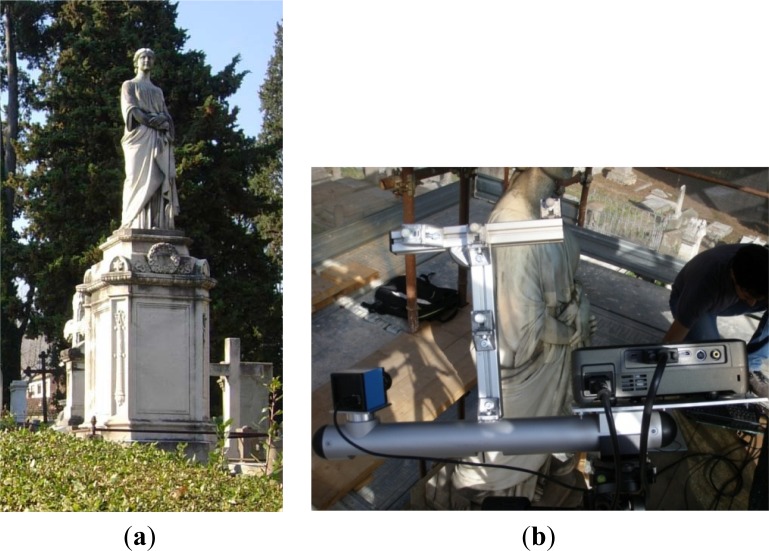
(**a**) The statue of Hope at the English Cemetery in Florence. (**b**) Acquisition activities around the statue.

The multi-sensor framework has been used to reconstruct the shape of a Statue of Hope (Figure 10) located at the English (Protestant) Cemetery of Florence (Italy). The statue is one of the earliest signed Statue of Hope memorials. It was carved by Odoardo Fantacchiotti in 1863 for the grave of Englishman Samuel Reginald Routh.

The optical acquisition of the Statue of Hope has shown up various critical aspects: (1) non controlled environmental lighting, compensated by a smooth variability of the surface color and a low level of brightness and contrast; (2) high variability of the local curvature of the shapes; (3) large extension of the surfaces to be measured (the statue is about 4 meters tall, including the basement). The reconstruction has regarded the acquisition of both the basement and the statue. The target object is enclosed within a bounding volume of 1.5 m × 1.5 m × 4 m (width × depth height).

The structured lighting scanner has been equipped with a high power digital projector, which provides fine tune functionalities in order to compensate disturbs due to the uncontrolled lighting conditions. The operator has tuned both lighting power and shutter time of the camera in order to capture well-contrasted images.

Moreover, the statue has presented a high variability of shape with a huge number of undercuts and steep slope changes in correspondence with the head and the folds of the dress, and small details over very large areas. Therefore, the field of view has been diversified by changing the optical set-up of the scanner in order to obtain the best compromise in terms of resolution, overall accuracy and file dimension. In particular, the field of view has been changed from 280 mm × 210 mm to 400 mm × 300 mm providing horizontal resolutions ranged from 0.28 mm to 0.4 mm, respectively, and measurement uncertainties from 0.03 mm to 0.05 mm, respectively.

**Figure 11.**
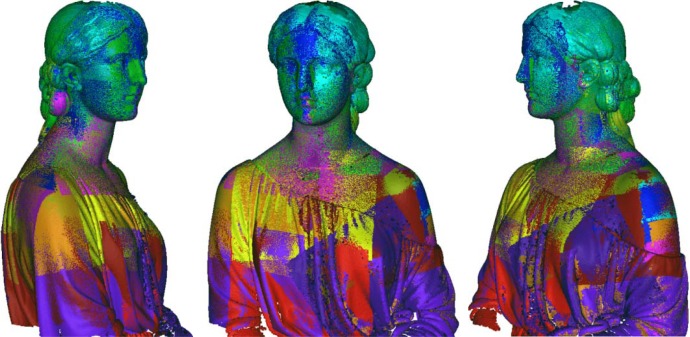
Aligned range maps of the upper part of the State of Hope.

**Figure 12.**
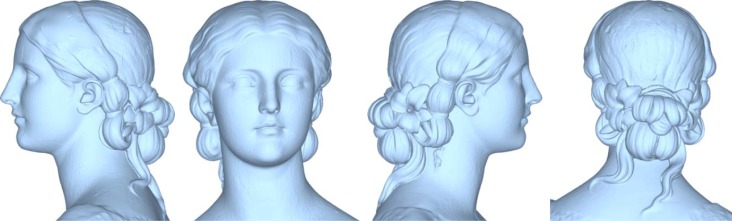
High resolution reconstruction of the statue head.

In the first phase of the work, the statue has been reconstructed capturing high resolution range maps of the upper side in order to obtain a high definition at the areas with formal details. The optical scanner has been set-up by decreasing the baseline in order to enhance the lateral resolution and to reduce the shadowing effects in the deepest sculptured areas. Figure 11 shows the alignment of 40 range maps including 12 million sample points by using the alignment procedure proposed in this paper. Once all the 3D range maps have been aligned with reference to the global coordinate system, they have been refined by Iterative Closest Point algorithms and merged to create a single polygonal mesh, which has been finally cleaned using editing tools. Figure 12 shows the reconstruction of the head, which has been accurately edited in order to use it for multimedia presentation and monitoring activities.

The optical configuration of structured lighting scanner has been changed for a larger field of view (lower resolution) to capture the other parts of the statue. This has involved the onsite calibration of the triangulation parameters of the optical system.

**Figure 13.**
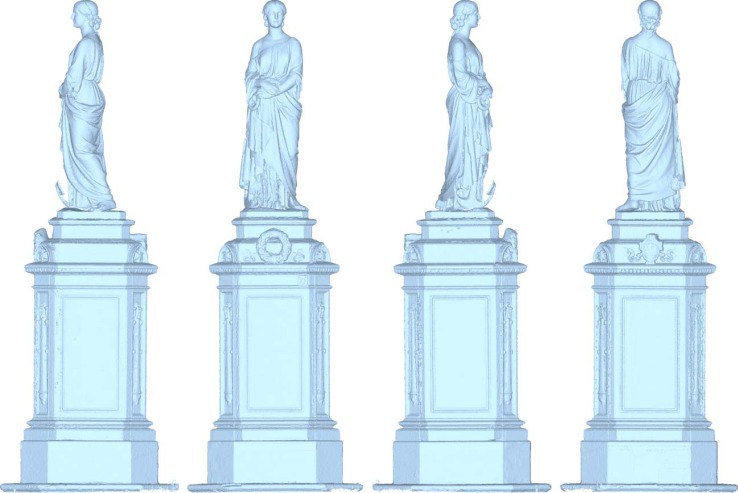
The complete 3D reconstruction of the Statue of Hope.

The overall target surface has been reconstructed using more than 200 range maps, which have been aligned by the optical tracking procedure and registered by a traditional ICP algorithm (Figure 13). In particular, the statue has been totally reconstructed by using three different locations of the passive stereo-photogrammetric device and moving the structured light sensors within the respective visible areas. A movement of the stereo-photogrammetric device has been tracked by keeping the scanner sensor in a fixed location. The frame of infrared (IR) active spherical markers has been measured from the two sequential locations of the stereo-photogrammetric device. This approach has allowed the registration of the groups of aligned range maps obtained by the respective stereo-photogrammetric device locations.

The measurements have provided a high level of robustness avoiding a degradation of the alignment accuracy due to the huge number of captured views. Moreover, the scanning process has not required a particular strategy for the range map alignment.

An experimental validation of the multi-sensor system cannot prescind from the environment conditions and the peculiarities of the target shape. A laboratory test would not have provided meaningful data to be extended to actual non cooperative contexts. In this work, the 3D reconstruction of the Statue of Hope has been verified by comparing the high resolution model with data captured using time of flight sensor Leica ScanStation [23]. In particular, the accuracy has been verified by comparing the relative distances between fully identifiable key points on the upper and bottom areas of the statue measured by both the time of flight sensor and the 3D scanning process. The comparison analysis has pointed out an average difference of 1.6 mm and a standard deviation of 1.4 mm. These values indicate that the two alternative optical technologies provide similar dimensional results. Of course, the multi-sensor system developed in this paper is able to provide a 3D reconstruction with a higher level of details than those obtainable by the time of flight sensor.

**Figure 14.**
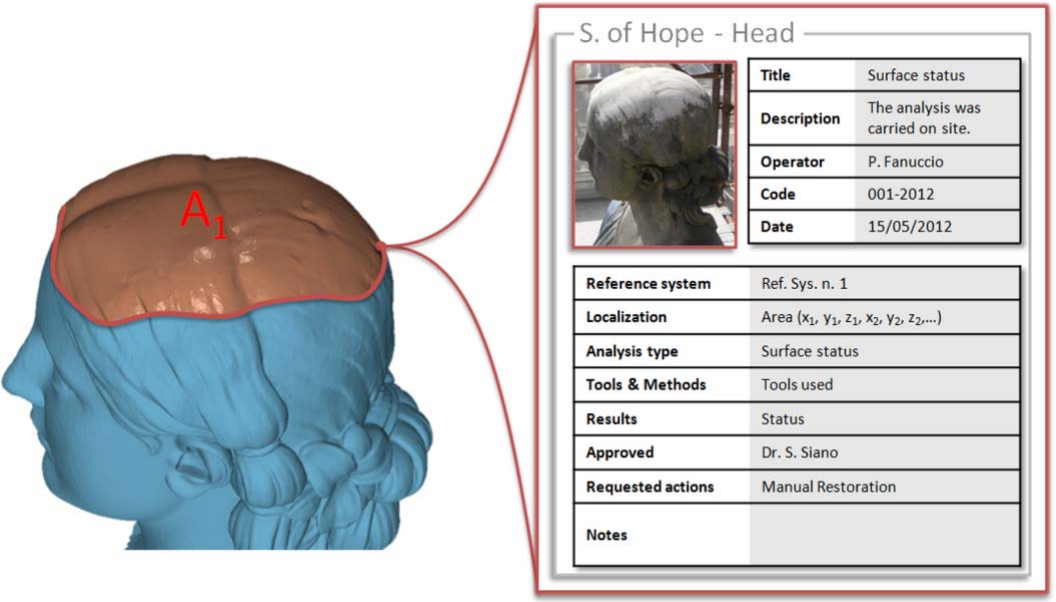
3D referring of metadata to the corresponding area of interest A_1_ partially covering the head of the Statue of Hope.

The complete representation has been also used to map information regarding the status of degradation. In particular, the stereo photogrammetric sensor has been used as independent stereo system to capture an area over the head of the statue. This area is affected by a degradation phenomenon due to rain water corrosion. The area has been defined by a point by point selection of the contour captured by the stereo image system. The corresponding 3D coordinates have been transformed into the artwork reference system through the rigid motion parameters. The 3D contour points have been orthogonally projected onto the artwork mesh model (Figure 14). The metadata relative to area dimensions and status of degradation have been stored in an open “*.xml*” file directly associated to the corresponding reference onto the 3D virtual model.

## 6. Conclusions

In this paper, a multi-sensor framework has been proposed to reconstruct three-dimensional virtual models of sculptural artworks. The method proposes the integration of two 3-D measurement sensors: a structured lighting scanner for high resolution measurement of range maps and a passive stereo photogrammetric sensor to track rigid motions of the scanner during multi-view processes. In particular, the methodology is based on monitoring the spatial position of a frame of active markers rigidly connected to the structured light scanner.

The methodology avoids accumulation errors, which typically occur in aligning a high number of range maps by standard pair-wise procedures, since high resolution range maps are always referred to the unique global coordinate system defined by stereo-photogrammetric sensor. Moreover, the tracker-driven alignment is an effective approach when external references cannot be used or overlapping areas between adjacent range maps are missing.

The methodology consists of a coarse alignment of the range maps captured by the scanner. The coarse alignment can be afterwards refined by downstream ICP procedures to distribute the misalignment error over the entire 3D model.

The digitalization methodology has been further integrated with a Reference Information System, which allows metadata related to testing and monitoring activities, to be directly addressed to 3D models. High-end representations of sculptural artworks can be progressively created by including any complementary information in the course of time.

In this paper, the methodology has been successfully applied to the virtual reconstruction of the Statue of Hope located at the English Cemetery in Florence, which has represented a severe test for the performance of the 3D digitalization technique. The target artwork exhibits large dimensions along with extremely complex features. The integration of an active structured light scanner and a passive stereo photogrammetric system has allowed the combination of the good local accuracy of the former with the extraordinary overall capability of the latter in managing a high number of range maps. The methodology has not required an exact planning of the scanning strategy at the beginning, but range maps have included in the overall reconstruction along the way.
